# Supplementary material for: The Evolution and Characterization of the RNA Interference Pathways in Lophotrochozoa
Source: Genome Biol Evol. 2024 May 7;16(5):evae098. doi: 10.1093/gbe/evae098 (PMC11114477; doi:10.1093/gbe/evae098)
Supplement: evae098_Supplementary_Data [file evae098_supplementary_data.zip › Supplementary_materials.zip!/selected_miRNAs/DaRe_selected_miRNas/DaRerGi_NC_007113.7_3175.pdf]

Provisional ID	:	NC_007113.7_3175
Score total	:	24.9
Score for star read(s)	:	3.9
Score for read counts	:	17.5
Score for mfe	:	2.5
Score for randfold	:	1.6
Score for cons. seed	:	-0.6
Total read count	:	46
Mature read count	:	35
Loop read count	:	0
Star read count	:	11

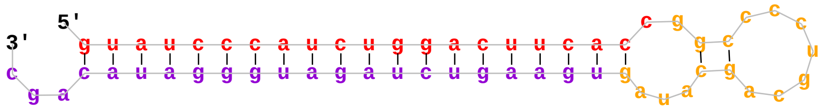

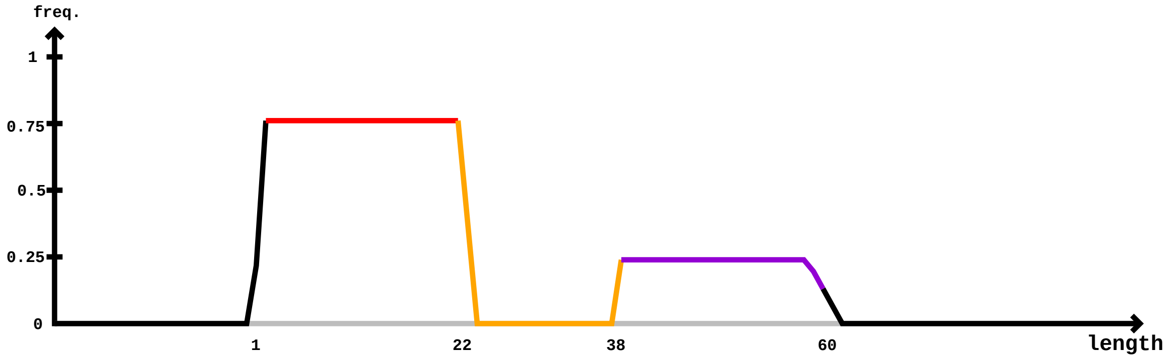

Mature Star

```
5'- ucucgugaguauccucagcuguaucccaucuggacuucaccggccccugcagcauagugaagucuagaugggauacagcagaggauacuagcgccaacauagcauacuuuu  -3'   obs
    ucucgugaguauccucagcuguaucccaucuggacuucaccggccccugcagcauagugaagucuagaugggauacagcagaggauacuagcgccaacauagcauacuuuu         exp
    ...(((.((((((((.((((((((((((((((((((((..((.......))...))))))))))))))))))))))).)))))))).)))................. reads  mm  sample
    ..................uguaucccaucuggacuucacc.......................................................................  2  0  Gu1
    ....................guaucccaucuggacuucacc......................................................................  11  0  Gu1
    .........................................................ugaGgucuagaugggauacagc................................  1  1  Gu1
    .........................................................ugaagucuagaugggauacagA................................  1  1  Gu1

    ....................guaucccaucuggacuucacc......................................................................  3  0  Ov3

    ..................uguaucccaucuggacuucacc.......................................................................  2  0  Gu2
    ....................guaucccaucuggacuucacc......................................................................  2  0  Gu2
    .........................................................ugaGgucuagaugggauaca..................................  2  1  Gu2
    .........................................................ugaGgucuagaugggauacag.................................  2  1  Gu2
    .........................................................ugaagucuagaugggauUcagc................................  2  1  Gu2

    ..................uguaucccaucuAgacuucacc.......................................................................  1  1  Ov2
    ..................uguaucccaucuggacuucacc.......................................................................  2  0  Ov2

    ....................guaucccaucuggacuucacc......................................................................  1  0  Ov1

    ..................uguaucccaucuggacuucacc.......................................................................  3  0  Gu3
    ....................guaucccaucuggacuucacc......................................................................  8  0  Gu3
    .........................................................ugaGgucuagaugggauacag.................................  1  1  Gu3
    .........................................................ugaGgucuagaugggauacagc................................  1  1  Gu3
    .........................................................Ggaagucuagaugggauacagc................................  1  1  Gu3
```
